# Biomimetic *In Vitro* Model of Canine Periodontal Ligament

**DOI:** 10.3390/ijms252212234

**Published:** 2024-11-14

**Authors:** Laura C. Pinho, José André Queirós, Catarina Santos, Bruno Colaço, Maria Helena Fernandes

**Affiliations:** 1 BoneLab—Laboratory for Bone Metabolism and Regeneration, Faculty of Dental Medicine, U.Porto, 4200-393 Porto, Portugal; laurapinho11@gmail.com; 2 LAQV/REQUIMTE, Faculty of Dental Medicine, U.Porto, 4200-393 Porto, Portugal; 3 CITAB—Centre for Research and Technology of Agro-Environmental and Biological Sciences, Inov4Agro, University of Trás-os-Montes and Alto Douro, 5000-801 Vila Real, Portugal; bcolaco@utad.pt; 4 CQE, IMS, Instituto Superior Técnico, University of Lisbon, 1049-001 Lisbon, Portugal; catarina.santos@estsetubal.ips.pt; 5 Instituto Politécnico de Setúbal, EST Setúbal, 2910-761 Setúbal, Portugal; 6 Hospital Veterinário Universitário de Paredes, 4580-593 Paredes, Portugal; andrequeiros5407@gmail.com; 7 CECAV–Animal and Veterinary Research Centre, University of Trás-os-Montes and Alto Douro, 5001-801 Vila Real, Portugal

**Keywords:** periodontal ligament, canine cell cultures, osteogenic induction, hydroxyapatite nanoparticles, osteoblastic markers, periodontal markers, biomimetic in vitro model

## Abstract

Periodontal disease affects about 80% of dogs, highlighting the importance of addressing periodontitis in veterinary dental care. The periodontal ligament (PDL) is a key structure holding the potential to regenerate the entire periodontal complex. This work presents an in vitro model of canine PDL-derived cell cultures that mimic the PDL’s regenerative capacity for both mineralised and soft tissues. Explant outgrowth-derived PDL cells were cultured under standard conditions in osteoinductive medium and with hydroxyapatite nanoparticles (Hap NPs). Cell behaviour was assessed for viability/proliferation, morphology, growth patterns, and the expression of osteogenic and periodontal markers. Osteogenic conditions, either achieved with osteoinducers or an osteoconductive biomaterial, strongly promoted PDL-derived cells’ commitment towards the osteogenic phenotype and significantly increased the expression of periodontal markers. These findings suggest that cultured PDL cells replicate the biological function of the PDL, supporting the regeneration of both soft and hard periodontal tissues under normal and demanding healing conditions. This in vitro model will offer a platform for testing new regenerative treatments and materials, ultimately contributing to canine dental care and better outcomes.

## 1. Introduction

Periodontal disease (PD) is among the most prevalent oral conditions in both humans [1] and companion animals. Notably, in dogs, the prevalence reaches around 80% by the age of 2 years [2]. Research findings suggest that the prevalence of PD can reach 100% when assessing dogs under anaesthesia or examining necropsy samples [3]. The disease is characterised by inflammation and progressive destruction of the periodontium, i.e., the tooth-supporting tissues (gingiva, periodontal ligament, alveolar bone, and root cementum), eventually leading to tooth loss. Even though preventive measures can be implemented, the aetiology, complexity [3,4], and lack of active dental care [5] contribute to the elevated risk of PD in dogs. Other aspects, such as age, breed, and size of the oral cavity, also contribute to the high prevalence of the disease [5,6]. Additionally, when left untreated, it can potentially contribute to systemic diseases [5]. This high incidence accounts for the significance of addressing periodontitis in veterinary dental care.

According to the American Veterinary Dental College, following stage one (gingivitis with no bone loss), early, established, and advanced periodontal disease in dogs is based on the percentage of bone loss around the teeth, respectively, <25%, 25% to 50%, and >50% [7]. Only the first stage can be reversed, and thus, the loss of alveolar bone remains the hallmark of periodontal disease progression [4,8], and its regeneration represents a therapeutic and clinical challenge. Besides infection control (mostly mechanical) and the establishment of an amenable repair environment, periodontal regenerative treatments are being increasingly considered to restore the function of the periodontium by exploring new biomaterials and tissue engineering strategies [9]. Most of the strategies focus on alveolar bone regeneration due to the role of this tissue in the disease outcome [10]. These studies are essentially being conducted in vivo, as reported in a recent review involving a large canine cohort of 112 Guided Tissue Regeneration (GTR) procedures performed from 2003–2021, suggesting that the available information is still insufficient to routinely incorporate this approach into veterinary clinical practice [9]. It should also be emphasised that dogs are considered an interesting animal model in preclinical therapeutic procedures due to their translational potential to human disorders, namely in the study of periodontal disease progression and the establishment of regenerative treatment protocols [11,12]. Further, due to this well-established knowledge, canine models were naturally transposed to implantology and validated as peri-implantitis models [13]. Unquestionably, the excessive use of animals, either in situations of naturally occurring disease or after disease induction, solely for research purposes, raises relevant ethical concerns [14,15] and underscores the need to resort to improved in vitro models, able to deliver relevant translation information to pursue the 3Rs policy to reduce the reliance on animal use [14,16]. 

In the periodontium complex, the periodontal ligament (PDL) stands as a key structure. It is a fibrous, soft, connective, and highly vascularised tissue connecting the alveolar bone (the bone that holds the tooth root) to the adjacent tooth cementum (hard tissue covering the tooth root). The PDL has an important role in supporting tooth function, maintaining homeostasis, and repairing damaged tissue in response to periodontal disease or mechanical trauma. As such, PDL encompasses a heterogeneous cell population that includes a small percentage of undifferentiated pluripotent mesenchymal stem cells holding the potential to regenerate the periodontal complex, being involved in the adult regeneration of the periodontal ligament, alveolar bone, and cementum [17,18]. In this regard, human PDL has been thoroughly studied as a source of mesenchymal stem cells able to differentiate in several cell types [19,20], anticipating their suitability in regenerative strategies [19]. The translational potential between humans and dogs has facilitated the examination of canine PDL-derived cells, also characterised by MSC traits [21,22]. However, studies dealing with canine PDL-derived cell cultures are sparse and typically entail comparison with human dental and other mesenchymal stem cells for translation to human research because of the similarity between canine dental tissue structure and pathophysiology to humans [23]. As such, the reported studies lack the specific focus of pursuing cellular and molecular representative and translational information able to address the canine periodontium pathophysiology and regenerative approaches. As mentioned above, these issues have been primarily studied in animal models and therefore with an emphasis primarily on regenerative outcomes. 

In this frame, the present work aims to report an in vitro model of canine PDL-derived cell cultures that would be representative of the biological role of the periodontal ligament, namely the ability to regenerate the mineralised tissues while keeping the competence to produce the periodontal ligament. Accordingly, initially, explant outgrowth-derived PDL cells were cultured under standard conditions and in osteoinductive medium to disclose their ability to differentiate into the osteogenic phenotype, aiming to mimic the potential to regenerate the supporting hard tissues. As such, cultures were characterised for viability/proliferation as well as molecular and cellular osteoblastic markers. As a novel added benefit, cultures were also evaluated for the expression of POSTN and S100A4, two classical periodontal markers, assessing their ability to replicate the potential for periodontal ligament regeneration. Following, as a proof-of-principle, explant-derived canine PDL cells cultured in standard conditions were exposed to hydroxyapatite nanoparticles (Hap NPs) due to their similarity to the inorganic matrix of the natural bone and also as a commonly investigated biomaterial for the regeneration of the periodontal hard tissues in humans [17,24,25] and dogs [26,27], with the cultures being characterised for NPs internalisation and osteogenic and periodontal markers. As such, this work aims to provide an in vitro model to address canine periodontal physiology, disease, and regenerative approaches. The goal is to reduce the use of animals in periodontal research and promote the ethical application of cell models. 

## 2. Results

### 2.1. Isolation of Periodontal Ligament-Derived Cells

**Figure 1:**
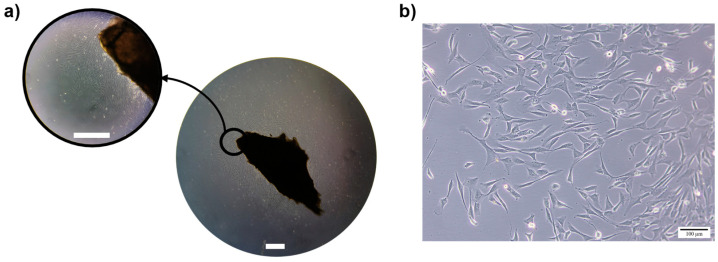
PDL-derived cells proliferating from the explant after 11 days of incubation (**a**) and after the medium exchange (**b**). Bar = 500 µm (**a**) and 100 µm (**b**).

Periodontal ligament (PDL)-derived cells were obtained by the explant culture method [28]. Cells’ outgrowth was visualised after 7–12 days of incubation (Figure 1a), and, following, cells presented a high growth rate. Under light microscopy, PDL-derived cells presented plastic adherence and a spindle/fibroblastic-shaped morphology (Figure 1b). Cell subculturing to expand the number of cells was performed until passage 3, being always accomplished at ~80% confluent to guarantee an exponential growth phase. At this stage, cells kept a high proliferative potential and an elongated morphology.

### 2.2. Behaviour of Periodontal Ligament-Derived Cells

PDL-derived cells were cultured in basal/growth medium and osteogenic-induced conditions (presence of ascorbic acid, dexamethasone, and β-glycerophosphate) for periods up to 21 days. Cultures were characterised for morphology, F-actin cytoskeleton, pattern of cell growth, viability/proliferation, and osteogenic and periodontal markers.

#### 2.2.1. Morphology and Cell Viability/Proliferation

**Figure 2:**
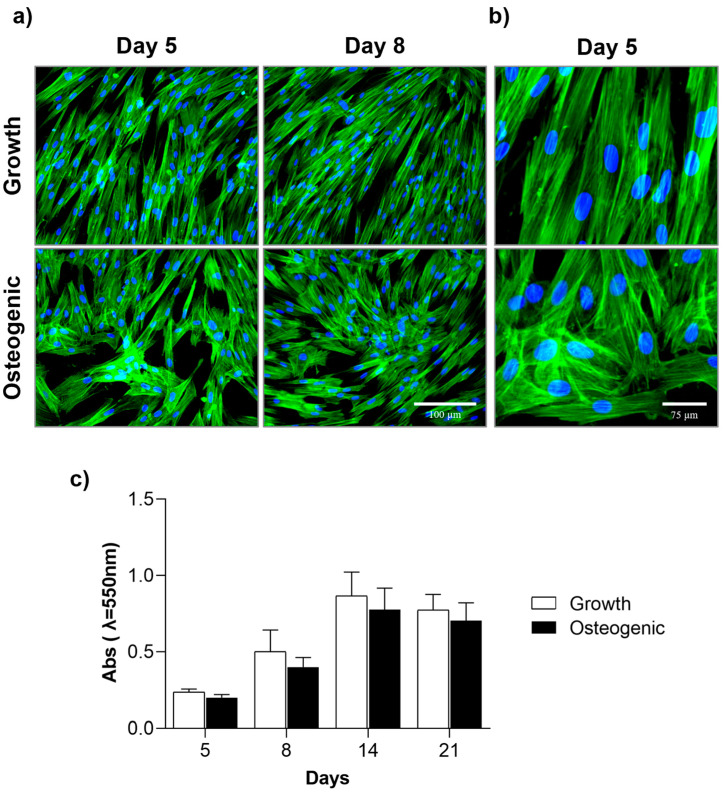
Immunocytochemical staining of F-actin cytoskeleton (green) and nucleus (blue) of uninduced (growth medium) and osteogenic-induced PDL-derived cell cultures on days 5 and 8; Bar = 100 µm (**a**) and 75 µm (**b**). (**c**) Metabolic Activity (MTT assay) of uninduced (growth medium) and osteogenic-induced cell cultures on days 5, 8, 14, and 21.

Cell morphology and the pattern of cell growth were clearly different in both culture conditions, as observed in the cultures immunostained for the F-actin cytoskeleton and nucleus (Figure 2a,b). Cultures kept in a growth medium displayed an ordered parallel-oriented pattern of cells with an elongated, fibroblast-like morphology, while osteogenic-induced cultures exhibited the presence of cellular groupments scattered on the cell layer; within the cell clusters, cells presented a different morphology showing an elongated-polygonal shape reflecting a higher expansion of the cytoplasm. These morphological features were well evident in the high-magnification images (Figure 2b). In both culture conditions, cells presented a well-organised F-actin cytoskeleton, and the changes in the cell morphology were accompanied by the rearrangement of this structure (Figure 2a,b).

Viability/proliferation was assessed by the MTT assay (Figure 2c). In both basal and osteogenic conditions, proliferation increased with the culture time, attaining maximal values around day 14 and decreasing slightly afterwards. Cell growth values were lower in the osteogenic medium, although without attaining statistical significance.

#### 2.2.2. Phenotype Differentiation: Gene Expression, SPP1 Immunostaining, ALP Activity

PDL-derived cells cultured in growth medium and osteogenic conditions were characterised by osteoblastic and periodontal cell markers.

**Figure 3:**
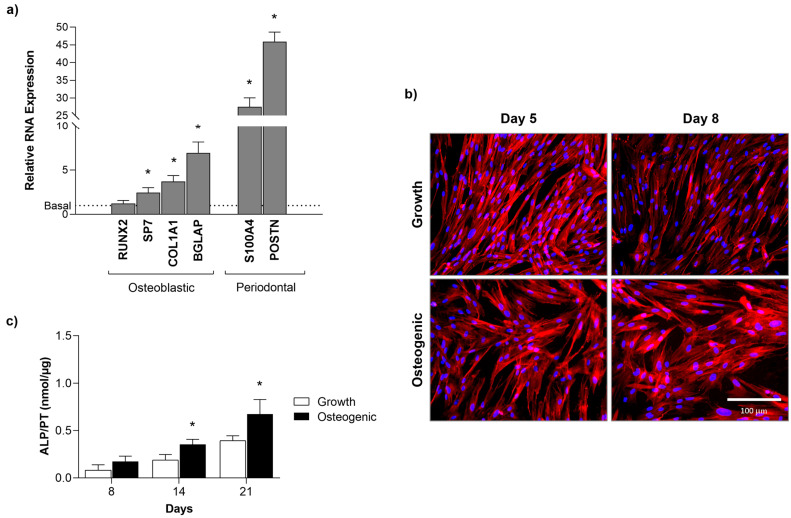
Differentiation of PDL-derived cells cultured in growth medium and osteogenic conditions. (**a**) Gene expression, at day 8; (**b**) Osteopontin immunostaining, at days 5 and 8, bar = 100 µm; (**c**) ALP activity, at days 8, 14, and 21. * Significantly different from the cultures performed in growth medium.

Gene expression by PDL cells growing in both culture conditions was evaluated at day 8 (Figure 3a), a phase of exponential cell growth as suggested by the results observed in the MTT assay. In this culture stage, cells cultured in a growth medium were able to express osteoblastic genes, i.e., RUNX2, SP7 (coding for osterix), Collagen a1 (coding for collagen type 1), BGLAP (coding for osteocalcin), and the periodontal-related genes S100A4 and POSTN. In osteogenic-induced conditions, the expression of RUNX2 was not affected, but there was a significant increase in the expression of all other genes. The highest induction was observed for the expression of BGLAP and the periodontal genes S100A4 and POSTN.

Cultures were also assessed for the presence of SPP1/osteopontin at days 5 and 8 (Figure 3b). Immunofluorescence images clearly show positive staining for this osteoblastic-related protein in both culture conditions, although with a higher intensity in the osteogenic-induced cultures. Images also validated the information concerning cell morphology and pattern of cell growth seen in the cultures stained for F-actin. The presence of cellular clustering in the osteogenic-induced cultures was well evident compared with that observed in the cultures performed in the growth medium. 

ALP activity was evaluated throughout the 21-day incubation time (Figure 3c). PDL cells incubated in a growth medium synthesise this enzyme, although the levels were significantly increased in osteogenic-induced conditions.

#### 2.2.3. Phenotype Differentiation: The Extracellular Matrix

**Figure 4:**
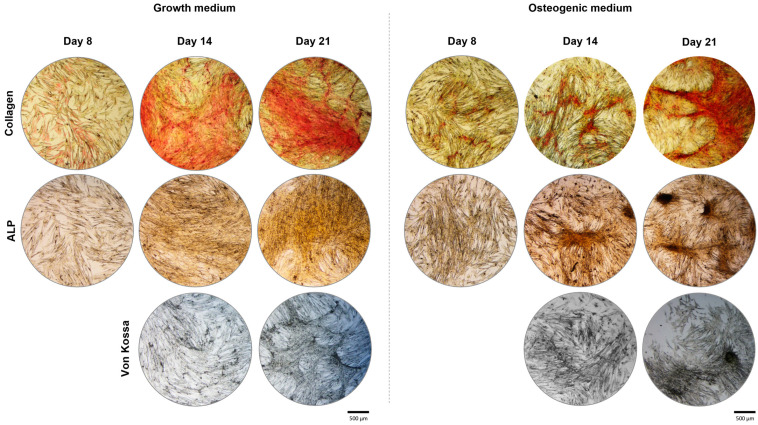
Differentiation of PDL-derived cells cultured in growth medium and osteogenic-induction conditions. Staining for Collagen (red), ALP (brown to black), and von Kossa (black) through the 21-day culture incubation. Bar = 500 µm.

The cellular layer of the PDL cell cultures performed in growth medium and osteogenic-induced conditions were stained for the presence of collagen, ALP, and mineralised calcium phosphate deposits (Figure 4). In addition, the stained cultures were allowed to follow the pattern of cell growth and organisation through the 21-day culture period in both culture conditions. 

PDL cell cultures maintained in a growth medium stained intensely for collagen and moderately for ALP. The cell layer increased through the incubation period along with a simultaneous increase in the staining intensity of both markers. Representative images suggest a slight tendency for a clustering pattern of the cell growth, especially noted from day 14 onwards. This was especially noticeable at day 21 in the von Kossa-stained cultures, which showed a weak tendency to form mineralised deposits.

PDL cells grown in osteogenic conditions presented a cell layer with a typical cell clustering organisation noticeable already at early culture times (day 8) and clearly evident on days 14 and 21. The cellular groupments exhibited a three-dimensional structure and stained intensively for collagen and ALP. Additionally, the cell clustering was associated with a positive reaction in the von Kossa assay, visible on day 21.

### 2.3. Proof-of-Principle: Responsiveness of PDL-Derived Cells to Hydroxyapatite Nanoparticles

#### 2.3.1. Physico-Chemical Characterization of Hydroxyapatite Nanoparticles

**Figure 5:**
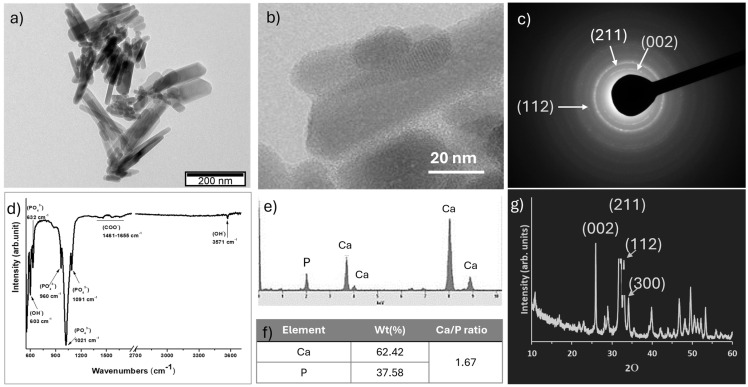
Transmission electron microscopy (TEM) (**a**,**b**) of developed Hap NPs with the corresponding electron diffraction pattern (SAED) (**c**); ATR-FTIR analysis (**d**) and chemical elemental composition determined by energy dispersive X-ray spectroscopy (EDX) (**e**) and inductively coupled plasma (ICP) (**f**); X-ray diffraction (XRD) diffractograms (**g**).

Hap nanoparticles were produced through a one-step hydrothermal process. TEM observation (Figure 5a,b) revealed that Hap NPs exhibit a rod-like morphology with an average length of 110 ± 10 nm and width of 25 ± 2 nm. The corresponding polycrystalline ring SAED pattern is presented in Figure 5c. The first ring in this SAED pattern corresponds to the (002) planes with a d-spacing of 0.344 nm, the strong second ring to the (211) planes, and the third ring to the (112) planes. Figure 5d shows the chemical groups of Hap NPs identified through ATR-FTIR spectra. The characteristic bands of phosphate groups are observed at 603 cm^−1^ for the bending vibration region and for stretching vibration at 960 cm^−1^, 1021 cm^−1^, and 1091 cm^−1^. Notably, the -OH group appeared at 3571 cm^−1^ and 632 cm^−1^ and belongs to -OH in Hap NPs. Additionally, the presence of –COO groups is confirmed by the peaks at 1461 cm^−1^, 1568 cm^−1^, and 1655 cm^−1^, indicating the existence of citrate. For a more in-depth examination of the chemical composition of the Hap NPs, EDX analyses were conducted. The presence of calcium (Ca) and phosphorus (P) in the Hap NPs was confirmed through EDX (Figure 5e), and the Ca/P atomic ratio of Hap NPs (Figure 5f) was found to be 1.66. The XRD pattern (Figure 5g) showed that the diffraction peaks of the Hap NPs closely corresponded to the standard reference pattern of Hydroxyapatite (JCPDS no. 09–0432), with no additional diffraction peaks being observed.

#### 2.3.2. Behaviour of PDL-Derived Cells Exposed to Hydroxyapatite Nanoparticles

PDL-derived cells cultured in a growth medium were exposed to hydroxyapatite nanoparticles (Hap NPs) (10, 50, and 100 µg/mL) for periods of up to 21 days. Cell behaviour was evaluated for morphology and F-actin cytoskeleton, pattern of cell growth, nanoparticles’ uptake, viability/proliferation, and phenotype differentiation.

**Figure 6:**
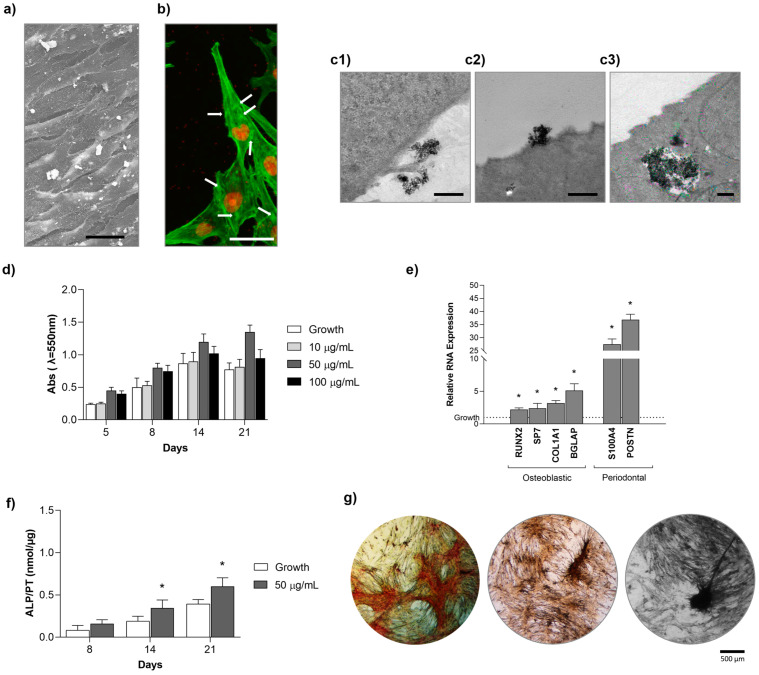
Behaviour of PDL-derived cell cultures exposed to hydroxyapatite nanoparticles for periods of up to 21 days. SEM image ((**a**), day 8, Bar = 30 μm), fluorescence image for F-actin (green) and nucleus (red) ((**b**), day 8, Bar = 75 µm), TEM images (**c**) showing the interaction of the cell surface with the Hap NPs ((**c1**,**c2**), Bar = 1 μm) and the NPs internalised and located in a large vacuole ((**c3**), Bar = 2 μm). Metabolic activity ((**d**), up to 21 days) of uninduced cultures (growth medium) and cultures exposed to hydroxyapatite nanoparticles. Phenotype differentiation, as assessed by the gene expression ((**e**), day 8), ALP activity ((**f**), up to 21 days), and histochemical staining of the cultures ((**g**), 21 days, Bar = 500 µm). * Significantly different from the cultures kept in growth medium (absence of Hap NPs).

Cells treated with 50 µg/mL Hap NPs were observed under SEM on day 8 (Figure 6a). At this culture time, cultures showed a parallel-oriented cell layer organisation without any signs of cytotoxic effects (intercellular gaps or retraction of the cell layer from the culture substratum); cells exhibited an elongated morphology, and the NPs were visible scattered in the cell layer and close association with the cell membrane. Fluorescence images of the cultures immunostained for the F-actin cytoskeleton and nucleus (Figure 6b) also align with a normal morphology and cytoskeleton organisation displaying parallel stress fibres and cell-to-cell intimate interactions. Additionally, the images suggested the presence of the particles inside the cells (Figure 6b, arrow). TEM observation of the cultures exposed to 50 µg/mL NPs for 24 h showed that the particles interacted closely with the cell membrane (Figure 6c1,c2) and were internalised being located in large vacuoles (Figure 6c3). Cells maintained normal organelles’ distribution in the absence of apoptotic or necrotic events, i.e., cell membrane shrinkage and bleb formation, cytoplasm budding, or chromatin condensation (Figure 6c3). Cell viability/proliferation, assessed by the MTT assay in the range of 10 to 100 µg/mL, was similar to the control (absence of NPs) through the 21-day culture time (Figure 6d). 

Phenotype differentiation of PDL-derived cells exposed to Hap NPs (50 µg/mL) revealed a significant increase in the expression of the tested osteoblastic and periodontal-related genes (Figure 6e, at day 8) as well as of ALP activity (Figure 6f, at days 8, 14, and 21). Further, at day 21, Hap-treated cultures exhibited a cell layer with a clustering organization. Staining for collagen, ALP, and mineralised deposits was mainly associated with the scattered cellular groups (Figure 6g, 21-day cultures).

## 3. Discussion

Periodontal disease is a prevalent dental health problem in dogs, affecting around 80% of adult animals [2,3,29]. As such, canine periodontal regeneration has been a very challenging field of research, having as a hallmark the use of animal models affected by naturally occurring or induced periodontal disease [8,30]. The use of in vitro models offers a significant advantage as they provide an ethical and scientifically valid alternative to traditional animal research [14,15]. Additionally, by being a closed system, they allow studying the involved cellular and molecular pathways. Nevertheless, there are also disadvantages due to the limited complexity of these models and extrapolation limitations [31]. Even though this work aims to provide a tool to analyse new regeneration strategies while creating awareness for the reduction of the number of used animals.

Having in mind that the regeneration of the alveolar bone is particularly relevant in periodontal regenerative strategies [9,24], existing research has provided valuable information concerning the stemness and osteogenic potential of cultured canine periodontal ligament-derived cells [21,22,32,33]. Accordingly, PDL-derived cell culture models have been mainly designed to address the regeneration of the hard tissues [34]. In this context, the present work represents a step forward in this vision. It describes an in vitro model showing that osteogenic differentiating canine PDL-derived cells also retain the ability to express typical markers of PDL, thus mimicking the in vivo role of the PDL in supporting the regeneration of hard (i.e., alveolar bone) as well as soft (i.e., periodontal ligament) tissues. 

In this work, canine PDL-derived cells grown from PDL explants in standard growth conditions presented surface (plastic) adhesiveness, fibroblastic appearance, and high proliferation rate, characteristic features of mesenchymal stromal cells. The primary culture showed cells with heterogeneous cell morphologies that became more homogenous after a few passages, which is in line with previous studies [22,23,32,35]. After, the differentiation studies were conducted in the third subculture that exhibited a homogeneous and well-organised layer of parallel-oriented fibroblastic-shaped cells. The responsiveness of canine PDL-derived cells was evaluated in osteogenic-induced conditions using the classic approach of supplementing the medium with dexamethasone, β-glycerophosphate, and ascorbic acid [36] and also by exposure to hydroxyapatite nanoparticles. In parallel, cultures kept in a standard growth medium (absence of inducers or Hap NPs) were assessed for the baseline behaviour. 

Canine PDL-derived cells cultured in a growth medium exhibited a high growth rate through the 21-day culture period. Although cultures showed mostly an organised layer of parallel-oriented and elongated cells, a slight tendency to form cellular groupments was noted, especially at longer culture times, i.e., at days 14 and 21. Histochemical staining revealed that cultures produced high levels of collagen, a major structural protein in the extracellular matrix crucial for tissue strength and integrity. Cultures also showed a positive reaction to the presence of ALP, an enzyme associated with osteoblast activity and with a key role in the onset of bone matrix mineralisation [37]. It should be noted that both components mostly present a relatively homogeneous distribution within the cell layer. Cultures produced SPP1/osteopontin, an early osteogenesis marker, and expressed key osteoblastic genes, i.e., RUNX2 and SP7 (the later coding for Osterix), which are transcription factors specific to early and late osteoblast development [38]. They also expressed Collagen a1 (coding for collagen type I, the main extracellular matrix component) and BGLAP (coding for osteocalcin, a non-collagenous protein marking later stages of osteoblast maturation). The described behaviour suggests a clear tendency of canine PDL-derived cells to differentiate towards the osteoblastic lineage in standard growing conditions. Despite the known pluripotency of PDL-derived cells [21], the basal commitment to an osteoblastic-like phenotype has long been documented in humans [39]. Nevertheless, in these in vitro basal conditions, canine PDL cells retain the ability to express genes characteristic of PDL, namely S100A4 and POSTN (the later coding for periostin), genetic markers associated with periodontal tissue repair and regeneration [22,35]. This behaviour might be related to the natural placement of PDL (between two hard tissues) and its site-specific functional role in preserving the structure and homeostasis of the periodontium. 

In the event of a diseased periodontium, such as that occurring in the periodontal disease, under the appropriate generated biological stimuli and healing demands, the periodontal ligament is recognised to be the structure in charge of regenerating the lost hard and soft periodontal tissues. Further, in most strategies, the regeneration of the alveolar bone is a hallmark due to its role in the disease outcome [18,40]. Accordingly, in the present work, canine PDL-derived cells were cultured in osteoinductive conditions to assess the representativeness of the model in mimicking the PDL feature of supporting the formation of the hard tissues. Compared to the cultures kept in a growth medium, canine PDL-derived cells cultured in osteogenic conditions formed a cell layer with a clear 3D clustering organisation seen already at early incubation time, with the cellular groupments staining intensively for collagen, ALP, and mineralised calcium phosphate deposits. This localised and intense staining pattern reflects a marked modulation of canine PDL-derived cells towards the osteogenic phenotype, in line with that seen previously [41]. The osteogenic conditions led also to a significant increase in the expression of classic osteogenic genes, namely SP7, collagen a1, and BGLAP. It is known that PDL-derived cells include a small proportion of mesenchymal stromal cells that can differentiate into an osteogenic phenotype in appropriate osteoinductive conditions, and this behaviour has been reported previously for humans [34,42] and also canine [23] cultured periodontal cells. However, additionally, the present work shows that canine PDL cells that are differentiating towards the osteogenic lineage also display a significantly upregulated expression of S100A4 and POSTN, about 30 and 50 times greater, respectively, compared to that observed in basal growth medium. These genes appear to play a critical role in periodontal regeneration, regulating both the formation of hard and soft tooth support tissues. Periostin (coded by POSTN) is an extracellular connective tissue marker preferentially expressed in collagen-rich fibrous connective tissues subjected to constant mechanical strains being present in tissues such as the periosteum, alveolar bone, and PDL. It appears to regulate cell migration, recruitment, adhesion, proliferation, and attachment to healing areas of various tissues. Thus, by promoting the migration of fibroblasts and osteoblasts, particularly at sites of hard-soft tissue interfaces, it might play an essential part in the maintenance and remodelling of the PDL and its surrounding bone [43]. Accordingly, periostin has been used as a successful periodontal regeneration marker [44]. As PDL remains unmineralized physiologically, it is thus thought that PDL cells possess mechanisms to inhibit mineralisation. S100A4 (also known as fibroblast-specific protein 1) is a calcium-binding protein synthesised and secreted by PDL cells and suggested to be involved in the response of human PDL cells to mechanical stress [45] and to be a negative regulator of PDL mineralisation and osteoblastic differentiation in human cells [35,46,47]. Therefore, periostin and S100A4 appear to be committed with distinct roles regarding the promotion/prevention of osteogenesis and matrix mineralisation, suggesting their relevance in achieving an optimal equilibrium regarding the regeneration of the lost periodontal hard and soft tissues. 

Hydroxyapatite is a material commonly used, alone or in combination, in periodontal regenerative strategies [48], including in canine periodontal disease [49]. Despite the promising applications of Hap NPs, there is a noticeable gap in studies investigating their effects specifically on canine PDL-derived cells in vitro. Having this in mind, as a proof-of-principle, canine PDL-derived cells cultured in standard growth medium were evaluated for their responsiveness to hydroxyapatite nanoparticles. Hap NPs are usually investigated due to their higher surface area-to-volume ratio compared to conventional Hap [26,27,49]. Hap NPs tested in this work were synthesised through a one-step hydrothermal process following a previously developed methodology [50,51,52]. The produced nanosized rod-like shaped NPs presented a Ca/P atomic ratio of 1.66, thus kept closer to that of bone hydroxyapatite (reported to be approximately 1.67) [53]. Further, the XRD pattern of the NPs indicates that Hap possesses a high-purity hexagonal crystalline structure [50,51], agreeing with the crystalised and hexagonal arrangement occurring in natural bone. ATR-FTIR, EDX, and ICP analysis showed the chemical features of Hap and, additionally, the presence of citrate [51]. This is an important component of the bone tissue (~5 wt%) known to participate in the biomineralisation process by the direct regulation of citrate-collagen interactions [54] and by stabilising Hap nanoparticles in bone [55]. 

Results for the exposure of canine PDL-derived cells to Hap NPs showed an intimate interaction of the particles with the cytoplasmic membrane, followed by their internalisation and location in cytoplasmic vesicles. Further, PDL-derived cells exposed to Hap NPs in a concentration that did not affect cell viability/proliferation and morphology organised into a typical clustered cellular layer, which was well evident in 21-day cultures. These 3D cellular clusters stained heavily for collagen, ALP, and mineralised deposits, suggesting the differentiation into the osteogenic phenotype. This was further supported by the significant induction in the expression of RUNX2, SP7, collagen a1, and BGLAP. These results agree with previous studies performed with these NPs interacting with osteoblastic MG63 cells [52] and human bone marrow mesenchymal stromal cells [50]. The presence of citrate ions might also have a positive contribution to the observed osteogenic induction of PDL-derived cells. It has been demonstrated that the release of citrate can promote the differentiation of human mesenchymal stem cells towards active bone-forming cells by regulating energy-producing metabolic pathways [56,57]. In general, the present results are in line with those reported for the interaction of Hap NPs with biological constituents [58] and those showing the safety and the osteogenic potential of Hap NPs in periodontal ligament stem cells [59]. They also align with in vivo studies describing positive bone formation outcomes in canine periodontal regeneration approaches involving the use of nanosized Hap, either alone or in combination with other treatments [26,27]. Most information on canine periodontal hard and soft tissue regeneration comes from studies in animals with either naturally occurring or experimentally induced periodontal disease. In this study, an in vitro system using canine PDL-derived cells demonstrated that these cells differentiate into an osteogenic phenotype under osteoinductive conditions, achieved either through chemical inducers or osteoconductive material. Notably, these osteoinductive environments also significantly enhance the expression of periodontal markers. This in vitro model, which simulates the role of PDL in regenerating both hard and soft tissues, fills a gap in current research. It is anticipated to be a valuable tool for studying the cellular and molecular mechanisms of periodontal tissue regeneration in both healthy and diseased states, potentially reducing reliance on animal use in canine periodontal research.

## 4. Materials and Methods

### 4.1. Isolation and Culture of Periodontal Ligament-Derived Cells

Teeth with intact periodontal ligament were collected from healthy animal cadavers donated for research purposes with the approval of the Ethical Committee of the University of Trás-os-Montes e Alto Douro under the reference Doc46-CE-UTAD-2022. Periodontal ligament (PDL)-derived cells were isolated from the middle third of the root of freshly extracted teeth from dog maxilla as described previously with minor modifications [28,41]. The periodontal ligament was carefully detached from the tooth using a scalpel, minced into small pieces, and the tissue fragments were plated as explants and cultured in standard growth medium (α-MEM supplemented with 10% foetal bovine serum, FBS, 100 IU/mL penicillin, 100 µg/mL streptomycin, and 2.5 µg/mL amphotericin B; all reagents from Gibco, Grand Island, NY, USA) at 37 °C in an atmosphere of 5% CO_2_ and 95% humidity. Cell outgrowth was evident after 7–10 days, and cultures were kept until optimal cell density was achieved (~80% confluency), with the culture medium being changed twice a week. Subculturing was then carried out during the exponential growth phase by enzymatically detaching the cell layer (0.04% trypsin, 37 °C, 10 min, Gibco, Grand Island, NY, USA) and transferring the resulting cell suspension into a fresh growth medium. Cells from the third subculture were used for the experiments.

### 4.2. Differentiation of Canine Periodontal Ligament-Derived Cells

Canine PDL-derived cells (third subculture) were cultured at a density of 10^4^ cells/cm^2^ in two experimental conditions: (i) growth medium (as described above) and (ii) osteogenic-induction conditions (growth medium supplemented with 50 µg/mL ascorbic acid, 10 nM β-glycerophosphate and 10 nM dexamethasone, all reagents from Sigma-Aldrich, St. Louis, MO, USA). Both cultures were grown for periods up to 21 days and characterized for viability/proliferation, morphology, F-actin cytoskeleton immunostaining, and osteogenic and periodontal phenotype markers.

### 4.3. Proof-of-Principle: Differentiation of PDL-Derived Cells Exposed to Hydroxyapatite Nanoparticles

PDL-derived cells were seeded at a density of 10^4^ cells/cm^2^ and cultured for 24 h for cell adhesion. Subsequently, cells cultured in a growth medium were exposed to Hap NPs (10, 50, and 100 µg/mL) for periods up to 21 days and characterised for viability/proliferation, internalisation of nanoparticles, and differentiation markers.

**Figure 7:**
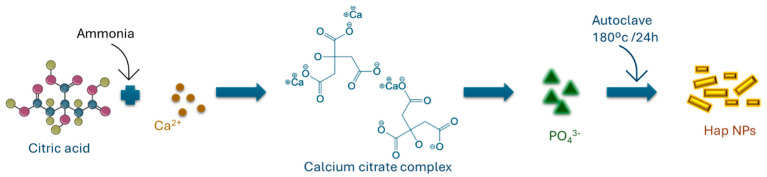
Schematic illustration of the synthesis of hydroxyapatite nanoparticles using citrate and a hydrothermal method.

Hap NPs were produced through a one-step hydrothermal process. The synthesis followed a methodology outlined in our prior research [50,51,52]. To summarise, an aqueous solution (0.6 M) of citric acid monohydrate (C_6_H_8_O_7_·H_2_O, 99.5%, Riedel-de-Haën), was prepared with a pH of 8.1 adjusted by adding 25% *v*/*v* ammonia (NH4OH, Riedel-de-Haën). Subsequently, calcium nitrate tetrahydrate (0.2 M), [Ca(NO_3_)_2_·4H_2_O, 99%, Riedel-de-Haën] was added to the citrate solution. Following this, a 0.2 M solution of (NH_4_)_2_HPO_4_ (Sigma-Aldrich, St. Louis, MO, USA) was slowly added dropwise while stirring. The resulting homogeneous solution was then transferred into a 50 mL Teflon-lined stainless-steel autoclave and maintained at 180 °C for 24 h. Finally, the precipitate was collected by filtration using a 0.22 μm Millipore filter and washed with Millipore water. Figure 7 presents a schematic illustration of the synthesis of Hap NPs using citrate and the hydrothermal method.

The size and the morphology of the synthesised Hap NPs were assessed by transmission electron microscopy (TEM) using a Hitachi H-8100-NA (Hitachi, Ltd., Tokyo, Japan) operating at an acceleration voltage of 200 kV. Prior to TEM analysis, Hap NPs were dispersed in ethanol using ultrasonication and deposited onto carbon-coated copper grids. Most Hap NPs were thin enough to be electron-transparent. Microdiffraction patterns were obtained in microdiffraction mode. The crystal structure of the Hap NPs was investigated using X-ray diffraction (XRD) on a Bruker D8 ADVANCE Powder Diffractometer, utilising monochromatic CuKα (target) radiation (1.5405 Å) at a scan rate of 0.02°/min. The calcium/phosphate ratio of the Hap NPs was analysed via inductively coupled plasma atomic emission spectrometry (ICP-AES) with instrumentation from Thermo Fisher Scientific (Waltham, MA, USA). Moreover, the main chemical groups present in Hap NPs were analysed using the attenuated total reflectance (ATR-FTIR) in transmittance, in the range of 600–4000 cm^−1^ using a Nicolet (Thermo Electron) spectrometer (Thermo Fisher Scientific, Waltham, MA, USA).

### 4.4. Characterization of PDL-Derived Cell Cultures

#### 4.4.1. Viability/Proliferation (MTT Assay)

The MTT assay was conducted during the 21-day culture period to evaluate the viability/proliferation of the PDL-derived cell cultures. MTT (5 mg/mL, Sigma-Aldrich, St. Louis, MO, USA) was added to the cultures for 3 h and incubated at 37 °C. The medium was removed, and dimethyl sulfoxide (DMSO, Sigma-Aldrich, St. Louis, MO, USA) was used (room temperature, 15 min) to dissolve the formazan salts characterised by their purple colour. Absorbance was measured at a wavelength of 550 nm in a microplate reader (Synergy HT, Biotek, Winooski, VT, USA).

#### 4.4.2. Alkaline Phosphatase (ALP) Activity

Cellular lysates from PDL-derived cell cultures were prepared by adding Triton X-100 (0.1%, Sigma-Aldrich, St. Louis, MO, USA) for 30 min. These lysates were used to assess alkaline phosphatase (ALP) levels and protein content. ALP activity was evaluated through the hydrolysis of p-nitrophenyl phosphate (p-NPP, 25 mM, Sigma-Aldrich, St. Louis, MO, USA) in an alkaline buffer (pH 10.3) for one hour at 37 °C. The reaction was terminated by adding NaOH 5 M (Sigma-Aldrich, St. Louis, MO, USA), and the resulting product, p-nitrophenol, was quantified at a wavelength of 400 nm using a microplate reader (Synergy HT, Biotek, Winooski, VT, USA). Results were normalised to total protein and determined using the DCTM Protein Assay (BioRad, Hercules, CA, USA) based on the manufacturer’s instructions. ALP activity was expressed as nanomoles of p-nitrophenol produced per microgram of protein (nmol/µg protein).

#### 4.4.3. Histochemical Staining Assays: Collagen, ALP, and Phosphate Deposits

Cell cultures were fixed (glutaraldehyde 1.5% in sodium cacodylate buffer 0.14 M, 15 min, TAAB, Aldermaston, Berks, England) and stained for the presence of collagen type I, ALP, and mineralised phosphate deposits.

Collagen staining: Fixed cultures (days 8, 14, and 21) were incubated with Sirius Red (1 h). Cultures were thoroughly washed with chloride acid 0.01N (Panreac, Barcelona, Spain) to remove the excess staining and left to dry for further observation; collagen deposits stained red. 

ALP staining: Fixed cultures (days 8, 14, and 21) were incubated in a filtered solution containing sodium naphthyl phosphate (2 mg/mL, Sigma-Aldrich, St. Louis, MO, USA) and Fast Blue RR in Tris buffer solution 0.1 M, pH 10 (2 mg/mL, Sigma-Aldrich, St. Louis, MO, USA) for 1 h protected from light and further observed for the characteristic brown-to-black staining. 

Phosphate deposits: Fixed cultures (days 14 and 21) were covered with a 1.0% silver nitrate solution (Sigma-Aldrich, St. Louis, MO, USA) and kept for 1 h under UV light. After, samples were washed with distilled water, and a solution of 5.0% sodium thiosulfate (Sigma-Aldrich, St. Louis, MO, USA) was added for 2 min. Cultures were then washed and left to dry for further observation. Von Kossa staining detects the presence of phosphate deposits, seen as black precipitates. 

Histochemically stained cultures were observed by light microscopy and image collection (Primo Vert™ Inverted Microscope, Carl Zeiss, Oberkochen, Germany).

#### 4.4.4. Immunostaining Assays: SPP1 (Osteopontin), F-Actin Cytoskeleton and Nucleus

PDL-derived cell cultures were fixed (formaldehyde 3.7%, 10 min, Sigma-Aldrich, St. Louis, MO, USA), permeabilized (Triton X-100 in PBS 0.1%, 30 min, room temperature), and incubated with bovine serum albumin (BSA, Sigma-Aldrich, St. Louis, MO, USA) in PBS 1% for 30 min (Sigma-Aldrich, St. Louis, MO, USA). For osteopontin staining, cultures were incubated overnight with the primary antibody, purified anti-osteopontin (SPP1) antibody (2.5 µg/mL, BioLegend, San Diego, CA, USA) and, after, with the secondary antibody, Alexa Fluor^®^ 594 Goat anti-mouse IgG (minimal x-reactivity) antibody (5 µg/mL, 2 h, BioLegend, San Diego, CA, USA). For F-actin cytoskeleton and nucleus staining, cells were incubated with Alexa Fluor^®^ 488 phalloidin (1:100, 30 min, Molecular Probes, Eugene, OR, USA) and Hoechst (8 µg/mL, 10 min, Enzo, Farmingdale, NY, USA). Images were obtained using the Celena S digital imaging system (Logos Biosystems, Anyang-si, Republic of Korea).

#### 4.4.5. Gene Expression by Real-Time Quantitative Polymerase Chain Reaction (RT-qPCR)

Expression of osteoblastic and periodontal genes was analysed by RT-qPCR on day 8. Total RNA was extracted using the TRIzol™ reagent (Invitrogen, Carlsbad, California, USA) and reverse-transcribed into complementary DNA (cDNA) with the NZY First-Strand cDNA Synthesis Kit (Nzytech, Lisbon, Portugal), according to the manufacturer’s instructions. Expression of the target genes (Table 1) was quantitatively determined on RT-PCR equipment (CFX96, Bio-Rad) using iQTM SYBR^®^ Green Supermix (BioRad, Hercules, CA, USA). 

#### 4.4.6. Cellular Uptake of Hydroxyapatite Nanoparticles

Cell cultures exposed to 50 μg/mL Hap NPs for 24 h were analysed for NPs’ internalisation by transmission electronic microscopy (TEM). Cultures were treated with a trypsin/EDTA solution to detach the adherent cells and centrifuged at 2000 g for 10 min. The resulting cell pellet was fixed (2.5% glutaraldehyde, 10 min), post-fixed (2% osmium tetroxide), and dehydrated through a graded ethanol series followed by a Spurr’s resin series. The cells were then embedded in a fresh 100% Spurr’s resin in moulds and polymerised at 60 °C for 8 h. Ultrathin sections (35–50 nm) were stained with 2% uranyl acetate and observed by TEM (JEOL 1200 EXII, JEOL, Akishima, Tokyo, Japan) at an acceleration voltage of 120 kV.

### 4.5. Statistical Analysis

Data were collected from three separate experiments, each conducted in triplicate, and are presented as mean values ± standard deviation. Statistical analysis was carried out using the JMP^®^ (7.0, JMP Statistical Discovery LLC, Cary, NC, USA) The *t*-test was used to compare experimental conditions, while one-way analysis of variance (ANOVA) was employed to compare multiple groups, followed by a post-hoc Tukey’s test. A *p*-value of ≤0.05 was considered statistically significant for both tests.

## 5. Conclusions

PDL-derived cells can differentiate into osteoblast-like cells under basal conditions while still expressing periodontal markers. Osteogenic conditions, either achieved with osteoinducers or an osteoconductive biomaterial, resulted in a strong commitment towards the osteogenic phenotype and a significant upregulation of periodontal markers. These findings suggest that cultured PDL-derived cells replicate the biological function of the PDL, supporting the regeneration of both soft and hard periodontal tissues under normal and demanding healing conditions. This in vitro model has the potential to be a useful tool for providing insights into the mechanisms underlying periodontal health and disease in canines. Additionally, it can serve as a platform for testing new regenerative treatments and materials, ultimately contributing to the advancement of canine dental care and improved outcomes.

## Figures and Tables

**Table 1:** Genes and respective primers assay ID (BioRad) for RT-qPCR.

Gene	Gene Name	Assay ID
Reference	Canis lupus familiaris actin, beta (ACTB)	qHsaCED0038674
Osteoblastic	Osteocalcin (BGLAP)	qCfaCED0031563
	Canis lupus familiaris collagen, type I, alpha 1 (COL1A1)	qCfaCED0027854
	Runt-related transcription factor 2 (RUNX2)	qCfaCED0033695
	Sp7 transcription factor (SP7)	qCfaCED0032017
Periodontal	S100 Calcium Binding Protein A4 (S100A4)	qCfaCED0027621
	Periostin (POSTN)	qCfaCED0035383

## Data Availability

The original contributions presented in this study are included in the article. Further inquiries can be directed to the corresponding author.
